# Early Retirement and Sensory Impairments: The Modifying Effect of Total Assets

**DOI:** 10.1093/geroni/igab046.1712

**Published:** 2021-12-17

**Authors:** Emmanuel Garcia Morales, Nicholas Reed

**Affiliations:** Johns Hopkins Bloomberg School of Public Health, Baltimore, Maryland, United States

## Abstract

Sensory impairments are common among older adults. Little is known on the association between sensory impairments, which impact labor productivity, and the effect modification of wealth. We used the 2006-2018 rounds of the Health and Retirement Study. Hearing (HI) and vision (VI) impairments (self-report) at baseline, and working status throughout the study period was observed. Logistic regression models, adjusted for demographic, socioeconomic, and health characteristics, were used to characterize the association of sensory impairment and early retirement (i.e., before age 65). Secondary analysis stratified by assets. Among 1,688 adults ages 53-64, 1,350 had no impairment, 140 had HI only, 141 VI only, and 57 had dual sensory impairment (DSI). Only adults with HI had higher odds of early retirement (Odds Ratio [OR]: 1.6; 95% Confidence Interval [CI]: 1.0,2.5) relative to those without sensory impairment. Among those with large assets, those with HI had higher odds (OR:2.6, 95% CI: 1.4,5.2) and those with VI had lower odds (OR. 0.37; 95% CI: 0.2,0.8) of early retirement. Among the low asset group, we found no differences across impairment groups for the odds of retirement. In sample of older adults, we provide evidence that the presence of hearing impairment is associated early retirement. Secondary analyses suggest wealth may modify this association which highlights the wealth disparities faced by people with sensory impairments.

